# Supplementary material for: Optimal Vegetable Intake for Metabolic-Dysfunction-Associated Steatotic Liver Disease (MASLD) Prevention: Insights from a South Italian Cohort
Source: Nutrients. 2025 Jul 29;17(15):2477. doi: 10.3390/nu17152477 (PMC12348136; doi:10.3390/nu17152477)
Supplement: Supplementary file 1 [file nutrients-17-02477-s001.zip › nutrients-3762216-supplementary.pdf]

Supplementary Materials

Liver Ultrasound

**Dimensions**	Normal	Increase	Reduced
**Margins**	Regular	Irregulars	
**Ecostructure**	Homogeneous Normoechogenic	Uneven	Hyperechogenic

Evaluation of Hepatic Steatosis

Contrast between liver parenchyma (P. EPA) and renal parenchyma (P. REN)	Deep beam penetration ultrasonic	Sharpness of the vascular structures, particularly particularly the veins
Homogeneous echo level and contrast between P. EPA and P. REN not evident (0)	Hepatic parenchyma clearly visible from the surface to the diaphragm (0)	Vascular structures clearly visible (0)
Slight discrepancy in echogenicity hepatic-renal (1)	Presence of opacity of the deeper parts of the liver or failure to visualize the diaphragm (1)	Loss of echoes of the vascular structures (1)
Wide discrepancy between hepatic and renal (2)	Presenza di opacità delle parti più profonde del fegato e mancata visualizzazione del diaframma (2)	Vascular structures not clearly visible (2)
**Steatosis score**: Steatosis Absent (0); Mild Steatosis (1-2); Moderate Steatosis (3-5); Severe Steatosis (6)		

Figure S1. Echographic sheet

Table S1. Collinearity Diagnostics of confounding variables

Variables:	VIF
Age	1.71
Gender	1.27
Education	1.56
Smoking	1.06
Daily kcal	2.77
γGT	1.20
AST/ALT	1.23
HOMA	1.17
Group Foods No Vegetable	2.17
Personal assessment of family income	1.08
Red Wine intake	1.20
Mean VIF	1.48

Variance inflation factors (VIFs), whether centred or uncentred, were calculated for the independent variables specified in the logistic model. A VIF is considered acceptable if it is less than 5.

Table S2. Micro and macro nutrients broken down by the presence or absence of MASLD

	MASLD		*p-value*[a]
	No	Yes	
N	668	629	
**Macronutrients:**			
Total Protein (g/day)	77.28 (26.18)	74.11 (27.82)	0.035
Animal Protein (g/day)	49.65 (20.42)	47.63 (20.17)	0.073
Vegetable Protein (g/day)	27.57 (10.37)	26.43 (11.39)	0.059
Total Lipids (g/day)	83.90 (31.66)	78.54 (31.78)	0.002
Animal Lipids (g/day)	41.20 (19.51)	38.01 (18.64)	0.003
Vegetable Lipids (g/day)	42.78 (18.30)	40.60 (18.62)	0.033
Saturated Totals (g/day)	26.43 (11.58)	24.31 (10.93)	<0.001
Total Monounsaturated (g/day)	39.15 (14.84)	36.90 (15.22)	0.007
Linoleic Acid (g/day)	8.91 (3.72)	8.42 (3.86)	0.020
Other Polyunsaturated (g/day	26.97 (55.96)	18.59 (37.39)	0.002
Total Polyunsaturated (g/day)	11.19 (4.41)	10.56 (4.57)	0.012
Cholesterol (g/day)	295.25 (132.52)	274.45 (122.72)	0.003
Glucides available (g/day)	258.52 (99.01)	244.53 (104.97)	0.014
Starch (g/day)	147.19 (67.23)	138.10 (72.00)	0.019
Soluble Glucides (g/day)	111.03 (51.44)	106.17 (50.65)	0.087
Dietary Fibre (g/day)	22.69 (7.99)	22.55 (8.83)	0.76
**Micronutrients:**			
Iron (mg/day)	11.14 (3.70)	10.70 (4.04)	0.044
Calcium (mg/day)	821.10 (401.04)	810.77 (382.62)	0.64
Sodium (mg/day)	2,149.26 (901.73)	2.039.16 (994.39)	0.037
Potassium (mg/day)	3,174.36 (1.033.57)	3,162.02 (1.090.35)	0.83
Phosphorus (mg/day)	1,284.16 (445.65)	1,250.42 (462.66)	0.18
Zinc (mg/day)	9.89 (3.30)	9.53 (3.57)	0.058
Thiamine (mg/day)	0.81 (0.29)	0.76 (0.30)	0.006
Riboflavin (mg/day)	1.23 (0.54)	1.18 (0.51)	0.098
Niacin (mg/day)	19.57 (7.18)	18.85 (6.98)	0.068
Ascorbic Acid (mg/day)	141.80 (66.73)	143.86 (71.43)	0.59
Pyridoxine (mg/day)	1.62 (0.57)	1.54 (0.58)	0.014
Folic Acid (mg/day)	294.12 (99.01)	282.41 (107.53)	0.041
Retinol (mg/day)	464.16 (528.66)	424.77 (402.61)	0.13
β Carotene (mg/day)	3,236.29 (1.625.18)	3,185.23 (1.802.44)	0.59
Vitamin E (mg/day)	13.49 (5.30)	13.01 (5.42)	0.11
Vitamin D (mg/day)	2.86 (1.52)	2.52 (1.37)	<0.001

[a]Wilcoxon rank-sum test. MASLD: Metabolic dysfunction-associated steatotic liver disease;
